# Ultrasound-Guided Clavipectoral Fascial Plane Block as a Sole Anaesthetic Technique for Clavicle Fracture Fixation: A Case Series

**DOI:** 10.7759/cureus.113883

**Published:** 2026-08-03

**Authors:** Rajesh Kumar, Sanjay Kumar, Poonam Kumari, Shashi Kant

**Affiliations:** 1 Anaesthesiology, All India Institute of Medical Sciences, Patna, Patna, IND; 2 Anaesthesiology (Trauma), All India Institute of Medical Sciences, Patna, Patna, IND

**Keywords:** clavicle fracture fixation, clavipectoral fascial plane block, high-risk perioperative management, phrenic-sparing anesthesia, ultrasound-guided regional anesthesia

## Abstract

The clavipectoral fascial plane block (CPB) is a new ultrasound-guided regional technique for clavicular surgery that spares phrenic nerve function, an important advantage over interscalene approaches. We describe three cases spanning very different risk profiles: a paediatric trauma patient, an octogenarian with significant pulmonary and renal disease, and a young trauma patient, all managed with clavicle fixation using CPB as the sole anaesthetic, without general anaesthesia. Each case proceeded with stable haemodynamics and no perioperative complications. These outcomes suggest CPB may offer a safer alternative for patients where phrenic nerve preservation or avoidance of general anaesthesia carries real clinical weight.

## Introduction

Clavicle fractures are common, and their surgical fixation has traditionally relied on general anaesthesia (GA) or interscalene brachial plexus block combined with superficial cervical plexus block [1]. Interscalene blockade reliably anaesthetises the clavicle but carries a near-universal risk of ipsilateral phrenic nerve block and hemidiaphragmatic paresis, a problem in patients with limited pulmonary reserve, paediatric patients, and trauma victims with thoracic injury [2]. GA adds the further risks of airway instrumentation and may be unavailable where resources are limited. The clavipectoral fascial plane block (CPB) is a comparatively recent ultrasound-guided technique that deposits local anaesthetic deep to the clavipectoral fascia, anaesthetising the supraclavicular, subclavian, lateral pectoral, and accessory nerve branches that supply the clavicle, while sparing the phrenic nerve [3]. Reports of CPB have grown steadily across diverse surgical populations [4]. We present three patients of markedly different ages and risk profiles in whom CPB served as the sole anaesthetic technique for clavicle fixation.

## Case presentation

Case 1

**Figure 1:**
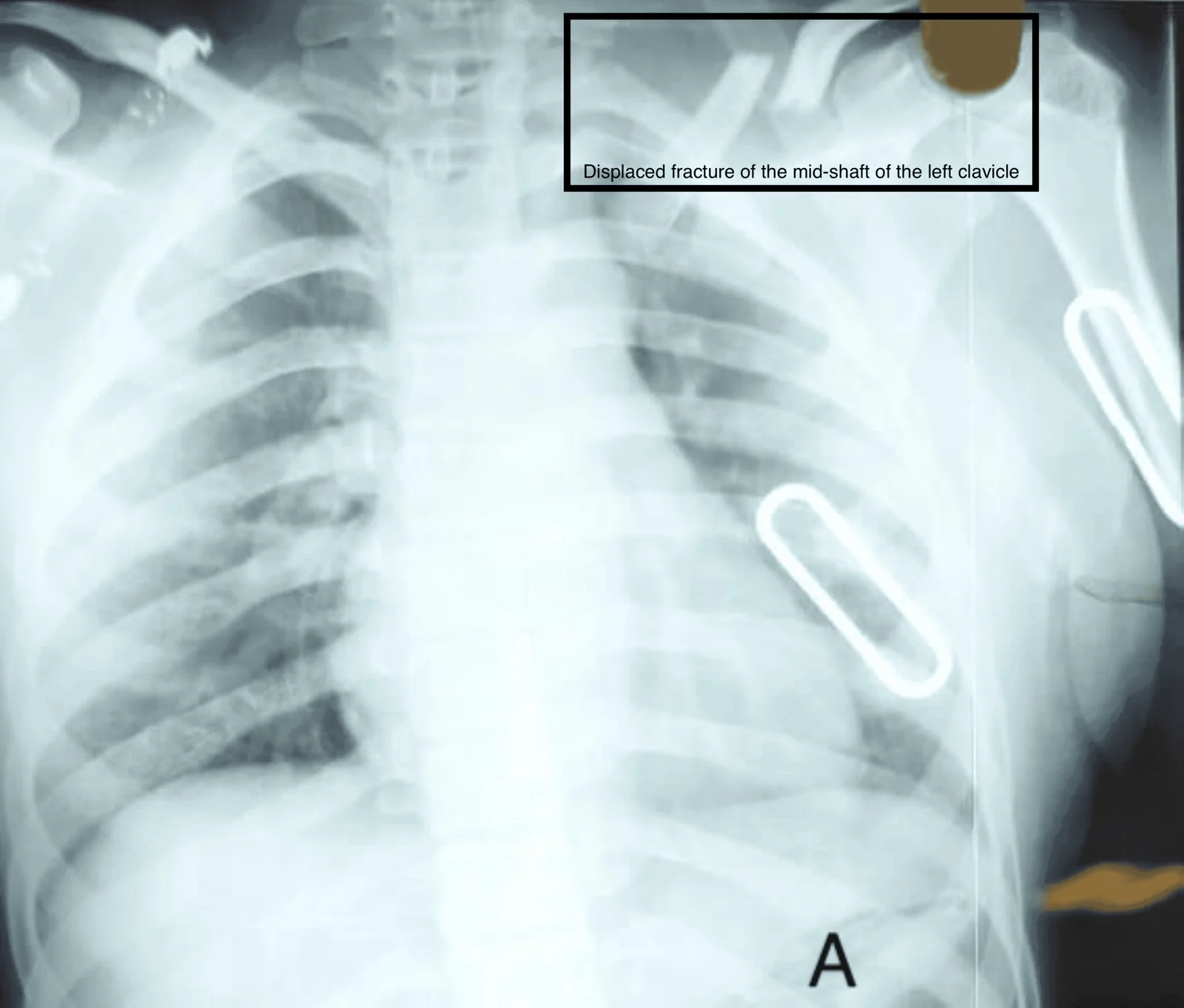
AP chest radiograph of an 18-year-old girl showing a displaced fracture of the mid-shaft of the left clavicle. AP: Anteroposterior

An 18-year-old girl sustained a displaced left midshaft clavicle fracture in a fall. Her parents declined GA on financial grounds. CPB was performed with 20 mL of 0.25% bupivacaine, split medially and laterally across the fracture line; sensory loss to pinprick was confirmed within 10 minutes. Open reduction and internal fixation (ORIF) took 70 minutes, with stable haemodynamics throughout and a visual analogue score (VAS) of 1-2/10 in the first postoperative hours (Figure 1).

Case 2

**Figure 2:**
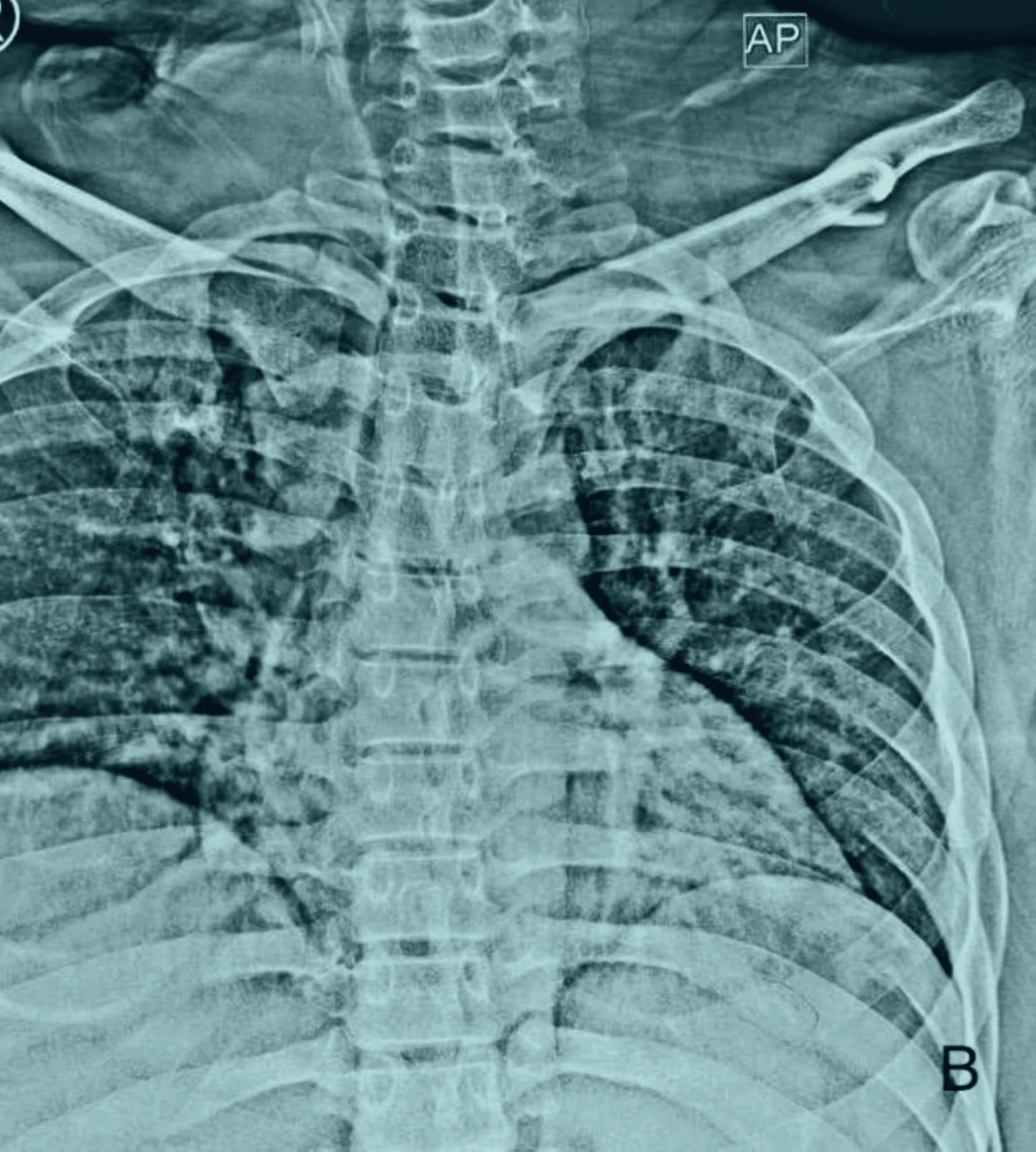
AP chest radiograph of an 85-year-old man with severe COPD showing a displaced fracture of the lateral third of the left clavicle. AP: anteroposterior; COPD: chronic obstructive pulmonary disease

An 85-year-old man with severe COPD (chronic obstructive pulmonary disease) on home CPAP (continuous positive airway pressure), type 2 diabetes, and end-stage renal disease on haemodialysis presented with a displaced lateral third of the left clavicle fracture. GA carried prohibitive risk given his pulmonary and renal status, so CPB was performed with 20 mL of 0.25% bupivacaine. ORIF proceeded over 60 minutes without sedation or supplemental analgesia; the patient reported a VAS of 0/10, with effective analgesia lasting up to 16 hours postoperatively (Figure 2).

Case 3

**Figure 3:**
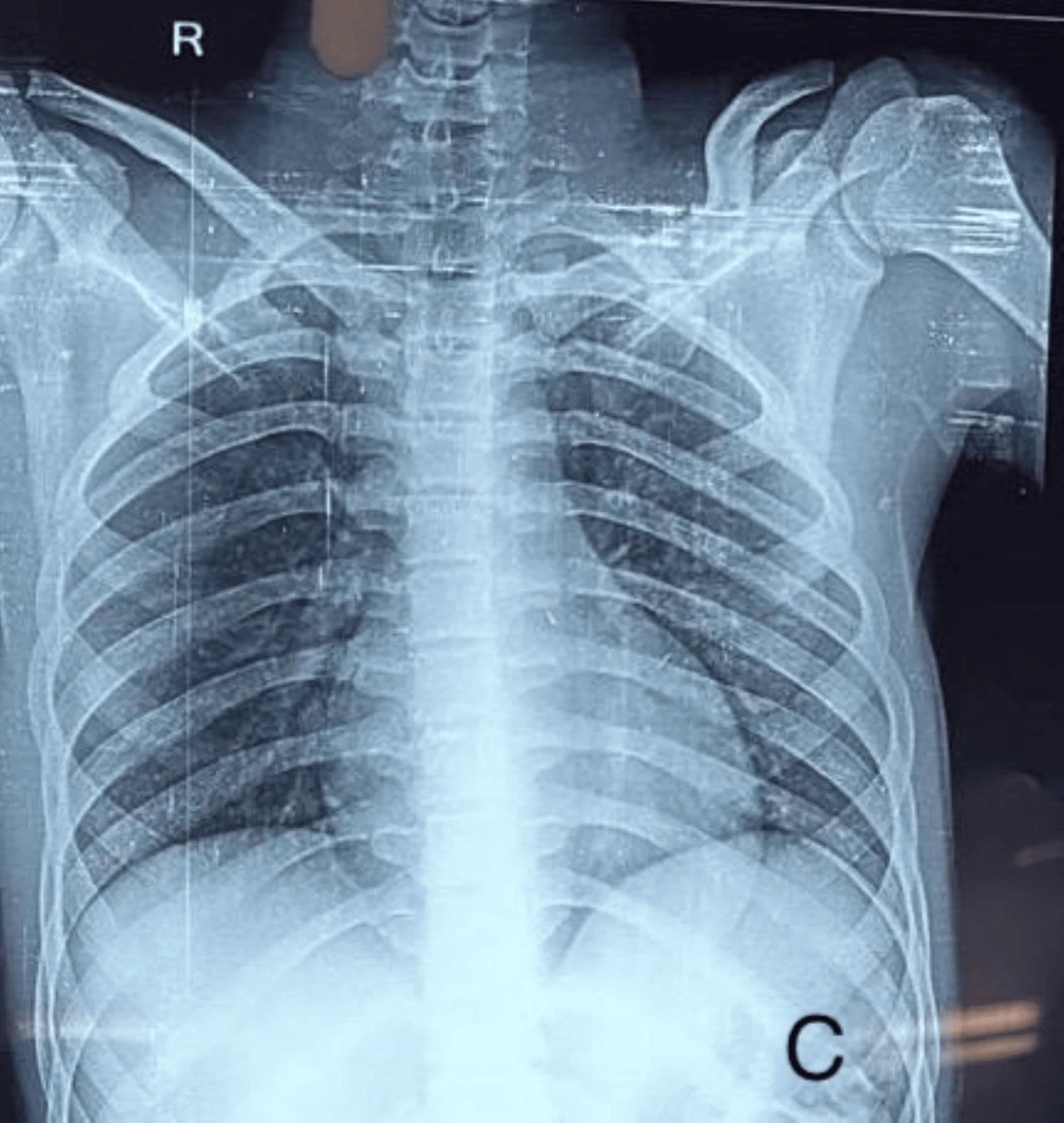
AP chest radiograph of a 19-year-old man showing a displaced fracture of the mid-shaft of the left clavicle. AP: anteroposterior

A 19-year-old man with an isolated left midshaft clavicle fracture, sustained in a road traffic collision, underwent CPB with 20 mL of 0.25% bupivacaine after his family refused GA. He remained awake and cooperative through 60 minutes of surgery, with VAS scores at or below 2/10 for eight hours postoperatively and no motor or respiratory compromise (Figure 3).

## Discussion

These three cases, spanning a vulnerable child, a multimorbid octogenarian, and a cooperative young adult, show that CPB can reliably deliver complete surgical anaesthesia for clavicle fixation without sedation, opioids, or GA, with stable intraoperative haemodynamics and postoperative analgesia lasting 8-16 hours. This pattern is consistent with the broader literature, which now includes case series, randomised trials, and a cadaveric anatomical study supporting the block's rationale [3-7].

The clinical case for CPB rests on its anatomy. Local anaesthetic deposited between the clavipectoral fascia and the periosteum bathes the terminal branches of the supraclavicular, subclavian, lateral pectoral, and accessory nerves, which together account for most of the clavicle's complex, dual-plexus innervation, while leaving the phrenic nerve and brachial plexus trunks untouched [3,7]. Randomised comparisons against superficial cervical plexus block and against combined interscalene-cervical plexus block have found CPB to be equivalent or superior in terms of pain scores, opioid consumption, and time to first rescue analgesia [5]. That respiratory-sparing profile mattered most in our COPD patient, where any hemidiaphragmatic paresis would have been poorly tolerated, and it is increasingly reported in paediatric and adolescent patients as well [4].

Beyond physiology, CPB is technically simple, requires only a linear probe, and can be performed in awake, cooperative patients, which made it usable here when families refused GA or when GA was financially out of reach, a genuine constraint in many trauma settings [8]. The series is small and uncontrolled, and it cannot establish optimal volume or concentration on its own; larger comparative studies, particularly in children, are still needed [6]. Even so, three successful cases across very different risk profiles argue that CPB deserves a routine place in the anaesthetist's options for clavicle surgery, not just a rescue role when GA is refused or contraindicated.

## Conclusions

CPB provided complete, phrenic-sparing surgical anaesthesia across three patients of contrasting age and risk, with stable haemodynamics and no complications. These findings support its consideration as a stand-alone technique for clavicle fixation, particularly when GA is a second choice, though larger controlled studies are needed to confirm optimal dosing and broader applicability.
